# The contribution of secondhand tobacco smoke to blood lead levels in US children and adolescents: a cross-sectional analysis of NHANES 2015–2018

**DOI:** 10.1186/s12889-023-16005-y

**Published:** 2023-06-13

**Authors:** Alexander Obeng, Taehyun Roh, Anisha Aggarwal, Kido Uyasmasi, Genny Carrillo

**Affiliations:** 1 Department of Environmental and Occupational Health, School of Public Health, Texas A&M University, College Station, TX 77843 USA; 2 Department of Epidemiology and Biostatistics, School of Public Health, Texas A&M University, College Station, TX 77843 USA; 3 Department of Health Behavior, School of Public Health, Texas A&M University, College Station, TX 77843 USA

**Keywords:** Lead exposure, Secondhand tobacco smoke, Blood lead level, Children and adolescents

## Abstract

**Background:**

Lead is a major developmental neurotoxicant in children, and tobacco smoke has been suggested as a source of lead exposure in vulnerable populations. This study evaluates the contribution of secondhand tobacco smoke (SHS) to blood lead levels (BLLs) in children and adolescents.

**Methods:**

We analyze data from 2,815 participants aged 6–19 years who participated in the National Health and Nutrition Examination Survey (2015–2018) to investigate the association between serum cotinine levels and BLLs. A multivariate linear regression was conducted to estimate geometric means (GMs) and the ratios of GMs after adjusting for all covariates.

**Results:**

The geometric means of BLLs in study participants aged 6 − 19 years were 0.46 µg/dl (95% CI 0.44, 0.49). After adjusting for relevant participant characteristics, the geometric means of BLLs were 18% (BLL 0.48 µg/dl, 95% CI 0.45, 0.51) and 29% (BLL 0.52 µg/dl, 95% CI 0.46, 0.59) higher in participants who had intermediate serum cotinine levels (0.03 − 3 ng/mL) and those who had high serum cotinine levels (> 3 ng/mL) respectively, compared to participants who had low serum cotinine levels (BLL 0.41 µg/dl, 95% CI 0.38, 0.43).

**Conclusions:**

SHS exposure may be a source of BLLs in US children and adolescents. Efforts to reduce lead exposure in children and adolescents should include strategies to reduce SHS exposure.

## Background

Lead is a neurotoxin that significantly damages young children and has no safe exposure level [1, 2]. Chronic exposure to environmental lead, even at relatively low levels, has been extensively documented to disrupt cognitive and motor functions, cause damage to the brain and peripheral organs, and promote obesity [3–5]. While remarkable progress has been made in reducing lead exposure in children in the US, including the Centers for Disease Control (CDC) recently lowering the reference dose of blood lead levels (BLLs) to 3.5 µg/dl based on recommendations by the Advisory Committee on Childhood Lead Poisoning Prevention (ACCLPP) [2], low-level exposure persists in millions of children [6].

The United States Environmental Protection Agency's (USEPA) federal action plan to reduce lead exposure and eliminate associated health impacts focuses specifically on reducing children's exposure to lead sources [7]. Child lead exposure in the United States has historically been attributable to: peeling and chipping lead-based paint, leaded gasoline, lead pipes, and smelting operations [8]. Other sources of lead exposure include children's jewelry, food items, batteries, glazed pottery, cosmetics, and medicinal folk remedies [8, 9]. The focus of national and local childhood lead prevention programs has changed from high levels to lower chronic levels of exposure; due to the declining average blood lead levels among US children [10]. This calls for considering potentially neglected sources of lead exposure, such as secondhand tobacco smoke (SHS).

Tobacco smoke contains more than seven thousand chemicals, including carcinogens and toxic heavy metals such as lead [11]. Exposure to tobacco smoke consists of mainstream smoke during puffing and SHS from a burning cigarette. Exposure to SHS poses a significant health concern in infants and children, including reduced lung growth, more frequent and severe asthma attacks, lower respiratory tract infections, and sudden infant death syndrome [12]. Children are primarily exposed to SHS by their parents and caregivers who smoke [13]. Approximately 20% of US children aged 3 − 11 years live with at least one active smoker [11], illustrating the importance of studying the relationship between SHS and child BLLs. Several studies have shown strong positive associations between active tobacco smoking and BLLs [14–16] However, very few studies have demonstrated the association between SHS and BLL [17–19], especially in children. Therefore, the present study aimed at evaluating the association between SHS exposure and blood lead levels (BLLs) in children and adolescents in the US.

## Methods

### Study design and population

This cross-sectional study was conducted to evaluate the relationship between SHS exposure (identified using serum cotinine levels) and BLLs in US children aged 6 − 19 years who participated in the NHANES between 2015 and 2018. The CDC's NHANES is a nationwide survey representing the general United States noninstitutionalized civilian resident population and includes comprehensive information on participants' health status, health behaviors, and sociodemographic characteristics. The NHANES used a complex stratified multistage probability design so that the sampled population accurately represents the general population of the US. We used two cycles of NHANES data (2015–2016 and 2017–2018) restricted to children and adolescents aged 6 − 19 with BLL measurements. Children three years or younger were excluded from this study because their serum cotinine was not tested. Participants with missing serum cotinine data and those with serum cotinine levels higher than 10 ng/mL (consistent with active smoking) [20] were excluded to ensure the sample contained nonsmokers only. The data from the most recent 2019 − 2020 cycle were excluded to prevent biased estimates because the data collection was disrupted in March 2020 due to the COVID-19 pandemic [21]. Among 4957 children and adolescents aged 6–19, 1,506 were excluded because their lead levels had not been measured. An additional 135 participants were excluded because their cotinine levels exceeded 10 ng/mL, and a further 501 subjects were excluded due to missing values for covariates. Finally, 2,815 children and adolescents were included in our analysis.

### Secondhand smoke exposure assessment

SHS was assessed using serum cotinine levels. Serum cotinine levels refer to the sum of cotinine and hydroxycotinine, as they are the primary metabolites of nicotine [22]. For this study, SHS exposure was categorized into 3 levels: low exposure (serum cotinine levels less than the limit of detection, 0.03 ng/mL), intermediate exposure (serum cotinine levels 0.03–3 ng/mL), and heavy exposure (serum cotinine levels > 3 ng/mL).

### Blood lead level measurement

All examined participants aged 1–11 years old and a one-half sample from participants aged 12 years and older were eligible for blood lead testing. Lead in whole blood was measured using inductively coupled plasma mass spectrometry (ICP-MS). The limit of detection (LOD) was 0.07 µg/dl, and concentrations below the detection limit were substituted with LOD divided by the square root of 2.

### QA/QC procedure

Blood collection occurred at an NHANES mobile examination center (MEC), where quality assurance and quality control procedures were carried out. These procedures were conducted not only in the MEC but also in contract and CDC laboratories responsible for measuring serum cotinine and blood lead levels (BLL). Comprehensive descriptions of these procedures have been provided in other studies [23].

### Covariates

Age, gender, central obesity, race/ethnicity (non-Hispanic White, non-Hispanic Black, Hispanic, other), and education of the household reference person (< high school, high school or some college, and ≥ college graduate) were included as covariates. Age was categorized into four groups (6–10, 11–15, and 16–19 years old). Central obesity was defined as having a ratio of waist circumference to height greater than 0.5, which is known to be a more predictive and reliable obesity indicator [22]. The family socioeconomic status was established by categorizing the poverty income ratio (PIR) as < 1.3, 1.3–3.5, and > 3.5. PIR is the ratio of the family’s income to its appropriate poverty threshold as defined by the US Census Bureau.

### Statistical analysis

SAS version 9.4 (SAS Institute, Inc, Cary, North Carolina) was used for statistical analyses to account for the complex sampling design and sampling weights in NHANES. Descriptive statistics, including frequencies, percentages, medians, and interquartile ranges of BLLs, were calculated for all levels of each variable, and the association between BLLs and other demographic characteristics was estimated. A Chi-square test was used to evaluate the relationship between the covariates, serum cotinine levels, and participant BLLs. Linear regression was performed to investigate the relationship between serum cotinine levels and BLLs was investigated using linear regression and the geometric means (GMs) and the ratios of GMs were estimated, adjusting for all covariates. The ratio of GMs was estimated by dividing the geometric mean of blood lead levels in a selected category by the geometric mean of blood lead levels in the reference category. Statistical significance was declared if a *p*-value was less than 0.05.

## Results

Table 1 shows serum cotinine level distribution among children and adolescents in the US. The percentage of participants with detectable serum cotinine levels (greater than 0.03 ng/ml) was highest in non-Hispanic Black participants, those with central obesity, those with less than high school education, and those with a poverty-to-income ratio of < 1.3. Among 2,815 participants, the median BLL increased with an increasing serum cotinine level.

**Table 1:** Characteristics of study population stratified by categories of serum cotinine levels: NHANES 2015–2018 (*n* = 2,815)

	**Serum cotinine Levels** Unweighted N (Weighted %)			***p*-value**^**c**^
	< 0.03	0.03–3	> 3 ng/ml	
**Total sample**	1153 (44.3)	1553 (52.3)	109 (3.4)	
**Gender**				0.313
Male	555 (43.1)	766 (52.9)	57 (4.0)	
Female	598 (5.5)	787 (51.6)	52 (2.9)	
**Age**				0.424
6 − 10	548 (45.8)	720 (50.9)	45 (3.3)	
11 − 15	382 (45.1)	537 (52.0)	33 (2.9)	
16 − 19	223 (40.7)	296 (54.84)	31 (4.5)	
**Race/ethnicity**				< 0.001
Non-Hispanic White	304 (43.9)	480 (52.2)	48 (3.9)	
Non-Hispanic Black	125 (21.9)	453 (73.3)	33 (4.8)	
Hispanic	331 (57.8)	241 (40.5)	8 (1.7)	
Others	393 (49.0)	379 (48.3)	20 (2.6)	
**Central Obesity**				0.0562
No	755 (46.5)	970 (50.4)	67 (3.1)	
Yes	398 (40.2)	583 (55.8)	42 (4.0)	
**HR Education**^**a**^				< 0.001
Less than High School	551 (37.4)	869 (57.7)	81 (4.9)	
High School or some college	431 (47.8)	571 (49.7)	27 (2.4)	
College or above	171 (63.3)	113 (36.3)	1 (0.4)	
**Poverty-to-income ratio**				< 0.001
< 1.3	324 (30.0)	713 (63.5)	74 (6.6)	
1.3 − 3.5	462 (41.3)	632 (55.9)	28 (2.8)	
> 3.5	367 (62.9)	208 (36.0)	7 (1.1)	
**Serum Cotinine Levels** Median (IQR^b^), ng/mL	0.02 (0.018, 0.024)	0.10 (0.05–0.33)	4.54 (3.69–6.29)	
**Blood Lead Levels** Median (IQR), ug/dl	0.39 (0.29–0.54)	0.48 (0.34–0.69)	0.54 (0.40–0.78)	

^a^Education, education of reference person in the household

^b^IQR interquartile range

^c^*p*-values based on chi-square test

**Fig. 1:**
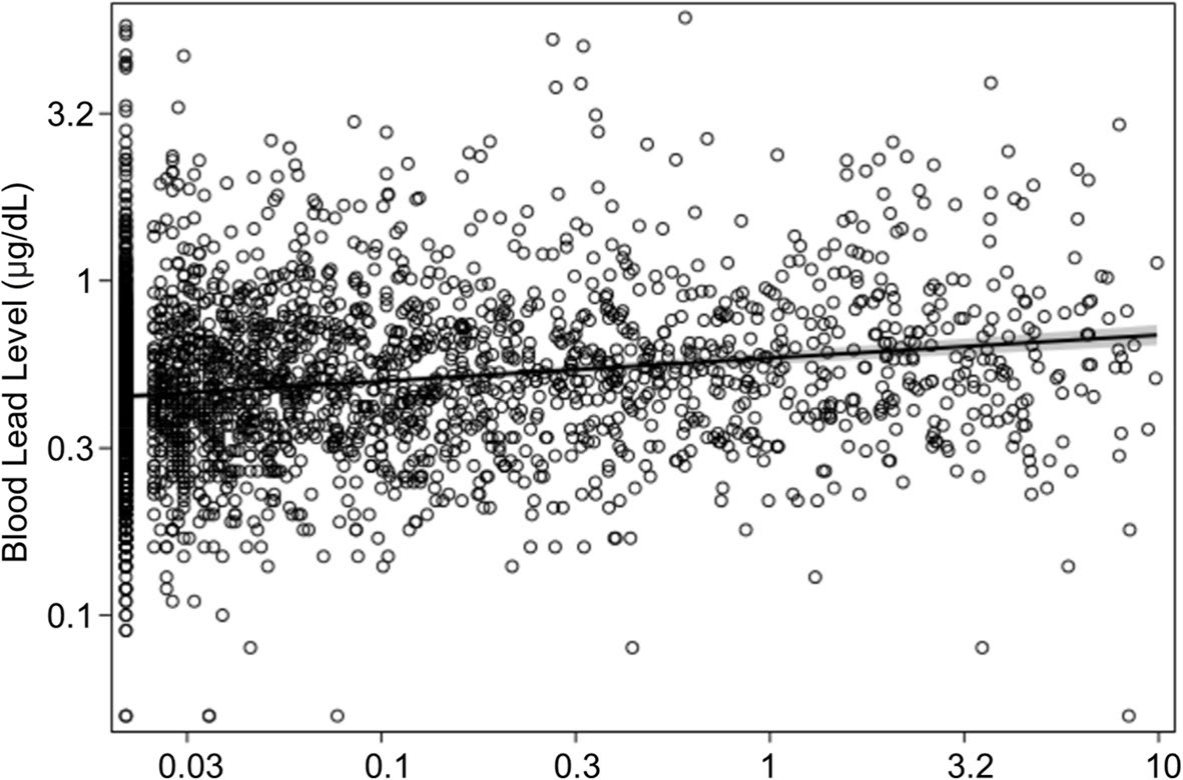
Log-scale scatter plot of serum cotinine and blood lead levels as continuous variables for all subjects (beta coefficient = 0.05, R-square = 0.11, *p* < 0.05). The bold line indicates a regression line, and the gray shade represents 95% confidence intervals

Figure 1 displays a log-scale scatter plot of serum cotinine and blood lead levels as continuous variables for all subjects, along with a regression line. The beta coefficient of 0.05 and R-square value of 0.11 indicated a weak positive association that was statistically significant (*p* < 0.01).

Table 2 compares the distribution of participants by categories of BLLs (≤ 0.45 or  > 0.45 µg/dl) across various sociodemographic characteristics. Significant differences were observed in the percentages of participants by categories of BLLs across most characteristics. The percentage of individuals with BLLs above the median of 0.45 µg/dl was highest in the 6–10-year age group (58.3%), and the percentages exhibited a declining trend with increasing age groups (*p*-value < 0.001). Additionally, males and non-Hispanic Black individuals had a higher proportion of participants with BLLs exceeding the median of 0.45 µg/dl. The percentage of participants with BLLs above the current reference value showed a decreasing trend with the increasing poverty-to-income ratio (*p*-value < 0.001), while no association was found across educational levels. Moreover, participants with heavier exposure to SHS had a higher percentage of individuals with BLLs above 0.45 µg/dl. Among 2,815 participants, 14 (0.4%) had BLLs above the new CDC blood lead reference value of 3.5 µg/dl.

**Table 2:** Percentage of subjects by categories of BLLs among US children and adolescents: NHANES 2015 − 2018

**Characteristics**	**Blood Lead Levels** Unweighted N (Weighted %)		***p*-value**^**b**^	**BLL > 3.5 µg/dl**^**c**^ Unweighted N (Weighted %)
	** ≤ 0.45 **^**a**^	** > 0.45 µg/dl**		
**Total sample**	1275 (50.0)	1540 (50.0)		14 (0.4)
**Gender**			< 0.001	
Male	522 (41.4)	856 (58.6)		4 (0.2)
Female	753 (58.5)	684 (41.5)		10 (0.5)
**Age group**			< 0.001	
6 − 10	489 (41.7)	824 (58.3)		5 (0.4)
11 − 15	453 (50.4)	499 (49.6)		5 (0.3)
16 − 19	333 (63.1)	217 (36.9)		4 (0.4)
**Race/ethnicity**			0.020	
Non-Hispanic White	377 (51.5)	455 (48.6)		3 (0.3)
Non-Hispanic Black	229 (40.4)	382 (59.6)		0 (0)
Hispanic	310 (55.5)	270 (44.5)		5 (0.8)
Other	359 (47.7)	433 (52.3)		6 (0.5)
**Central Obesity**			< 0.001	
No	739 (46.4)	1053 (53.5)		10 (0.4)
Yes	536 (56.7)	487 (43.3)		4 (0.3)
**Education**			0.271	
Less than a high school	725 (52.1)	776 (47.9)		9 (0.5)
High school or some college	425 (48.1)	604 (51.9)		5 (0.3)
College graduate or above	125 (47.0)	160 (53.0)		0 (0)
**Poverty Income Ratio**			< 0.001	
< 1.3	457 (43.6)	654 (56.4)		7 (0.5)
1.3–3.5	508 (47.5)	614 (52.5)		6 (0.5)
> 3.5	310 (59.8)	272 (40.2)		1 (0.1)
**Serum Cotinine levels**			< 0.001	
< 0.03 ng/mL	636 (59.7)	517 (40.3)		7 (0.3)
0.03–3	606 (43.1)	947 (56.9)		6 (0.3)
> 3	33 (30.2)	76 (69.8)		1 (1.0)

^a^Median of blood lead level among the entire study population;

^b^*p*-values derived from chi-square test comparing subjects with BLL below and above the median (0.45 µg/dl) across characteristics

^c^CDC's blood lead reference value

Table 3 shows the geometric means (GMs) and the ratio of GMs of BLLs from linear regression models for their associations with serum cotinine levels. After adjusting for relevant participant characteristics, participants with serum cotinine levels between 0.03 and 3 ng/mL (intermediate exposure) and greater than 3 ng/mL (heavy exposure) had 18% (0.48 µg/dl, 95% CI 0.45, 0.51) and 29% (0.52 µg/dl, 95% CI 0.46, 0.59) significantly higher BLLs than those who had levels less than 0.03 ng/mL (low exposure) (0.41 µg/dl, 95% CI 0.38, 0.43). A significant dose–response relationship was found between serum cotinine levels and BLLs (*p*-trend < 0.001). Results of the linear regression also showed that participants older than 11 to 19 years have between 11 to 21% lower BLLs than those who are 6–10 years. The GMs of BLLs were significantly higher in males, while Hispanic participants and those with central obesity showed significantly lower BLLs. The BLLs also decreased with increasing poverty-to-income ratio (*p*-trend < 0.001).

**Table 3:** Adjusted^a^ geometric means of BLL and ratio of GMs among US children and adolescents

**Characteristics**	**Blood Lead Level, μg/dL (GMs, 95%)**	**Ratio of GMs** **(95% CI)**	***p*-value**	***p*-trend**^***b***^
**Total**	0.46 (0.44, 0.49)			
**Serum cotinine levels**				< 0.001
< 0.03 ng/mL	0.41 (0.38, 0.43)	1.00		
0.03–3	0.48 (0.45, 0.51)	1.18 (1.12, 1.24)	< 0.001	
> 3	0.52 (0.46, 0.59)	1.29 (1.13, 1.46)	< 0.001	
**Gender**				< 0.001
Female	0.43 (0.40, 0.45)	1.00		
Male	0.51 (0.48, 0.55)	1.20 (1.15, 1.26)	< 0.001	
**Age**				
6–10	0.52 (0.50, 0.55)	1.00		< 0.001
11–15	0.47 (0.44, 0.50)	0.89 (0.84, 0.95)	< 0.001	
16–19	0.41 (0.38, 0.45)	0.79 (0.73, 0.85)	< 0.001	
**Race/ethnicity**				0.264
Non-Hispanic White	0.49 (0.45, 0.53)	1.00		
Non-Hispanic Black	0.47 (0.44, 0.51)	0.97 (0.88, 1.06)	0.433	
Hispanic	0.44 (0.40, 0.48)	0.90 (0.82, 0.99)	0.031	
Other	0.47 (0.43, 0.51)	0.96 (0.86, 1.07)	0.455	
**Central Obesity**				< 0.001
No	0.49 (0.46, 0.52)	1.00		
Yes	0.44 (0.41, 0.47)	0.90 (0.86, 0.94)	< 0.001	
**Education**				0.215
Less than High School	0.45 (0.43, 0.47)	1.00		
High school or some college	0.47 (0.44, 0.50)	1.04 (0.97, 1.12)	0.084	
College graduate or above	0.49 (0.43, 0.55)	1.08 (0.94, 1.25)	0.057	
**Poverty Income Ratio**				< 0.001
< 1.3	0.52 (0.48, 0.56)	1.00		
1.3–3.5	0.48 (0.45, 0.51)	0.92 (0.87, 0.97)	< 0.001	
> 3.5	0.41 (0.38, 0.44)	0.79 (0.73, 0.86)	< 0.001	

^a^Adjusted for gender, age, race/ethnicity, central obesity, education of reference person in the household, and poverty-to-income ratio*;*

^b^*p* values for trend were estimated to assess dose–response effect across categories of serum cotinine levels in the regression models

## Discussion

The present study aimed to evaluate the relationship between SHS exposure (identified using serum cotinine level) and BLLs in US children aged 6 to 19 who participated in the NHANES 2015 − 2018 and found the BLLs were 18% (BLLs 0.48 µg/dl, 95% CI 0.45, 0.51) and 29% (BLLs 0.52 µg/dl, 95% CI 0.46, 0.59) higher in study participants who had intermediate serum cotinine levels (0.03 − 3 ng/mL) and those who had high serum cotinine levels (> 3 ng/mL) respectively, compared to participants who had low serum cotinine levels (BLL 0.41 µg/dl, 95% CI 0.38, 0.43) in a significant dose–response manner. These findings supplement previous evidence for a potential association between SHS and high BLLs among US children [17, 19, 24]. Our findings and evidence from previous studies provide compelling grounds for strategies focused on reducing SHS exposure when designing children's prevention programs.

Initial examination of our results showed that the mean BLLs were lowest in Hispanic participants. A study by Teye et al. showed that children of Hispanic origin had similar BLLs compared to non-Hispanic whites. However, when adjusted for education level and income, Hispanic children had a significantly lowered BLL than non-Hispanic White children [25]. Those results highlight the importance of the socioeconomic status in lead exposure among children. Cornelius et al. also reported that in 2020, 8.0% of adults in families of Hispanic origin smoke cigarettes when compared with non-Hispanic Whites (13.3%), non-Hispanic blacks (14.4%), and Others (19.5%) [26]. This could explain the lower mean BLLs observed in children of Hispanic origin. Our results are consistent with an EPA analysis of CDC data from 1999–2015. They observed that non-Hispanic Black children and adolescents in the US have the highest average BLLs compared to their non-Hispanic white and Hispanic counterparts [27]. Similar to results from other peer-reviewed literature [27–29] is our finding that males, on average, have significantly higher BLLs than females, which is especially worrying since studies have also shown that males are more susceptible to the effects of lead than females [27, 28, 30].

Our study reports a significantly lower BLL among children from families with higher socioeconomic status (PIR > 3.5: BLL = 0.41 μg/dL, 95% CI 0.38, 0.43). One possible reason for this outcome could be the elevated smoking rates observed among individuals from low-income households. Additionally, children from families with lower socioeconomic status may encounter other sources of lead exposure, such as deteriorating paint in older homes. For example, Cornelius et al. reported that 20.2% of adults smoked cigarettes in households with lower income (Annual Household income < $35,000) vs. 6.2% of adult cigarette smokers from higher-income households (Annual Household income ≥ $100,000) [26]. Comparing our findings to the new CDC blood lead guidelines, 0.4% of our study participants had BLLs ≥ 3.5 µg/dl. This is lower than the expected 2.5% of participants in a previous study [2] mainly because the blood lead reference value analysis included only children from 1 to 5 years old. Our study excluded children younger than 6 years due to missing serum cotinine measurements and restricted availability of blood lead level data for children aged 3–5 years. Consequently, our study only included children older than 6 years up to 19 years. It also showed that the highest percentage of participants with BLLs above the median, 0.48 μg/dL were children aged 6 − 10. These results follow trends similar to an EPA report that states that younger children have the highest BLLs compared to their older counterparts [27].

We found that children aged 6 − 10 had the highest average BLL compared to all other age groups. Multiple factors can be attributed to this pattern. The first factor that may be contributing to the higher average BLLs among children in this age group is their behavioral patterns, such as hand-to-mouth activities where chemicals are transferred from soil or contaminated surfaces to hands and then ingested. The EPA presented that the frequency of hand-to-mouth behavior among children is inversely related to the age of the children [31, 32]. Among children aged 6–10, the average frequency of indoor hand-to-mouth contact was 6.7 times per hour, while outdoor hand-to-mouth contact occurred at an average frequency of 2.9 times per hour [32]. Similarly, the average frequency of indoor object-to-mouth contact was 1.1 times per hour, and outdoor object-to-mouth contact was observed at an average frequency of 1.9 times per hour in the same age group [33]. As the child's age increases, the frequency of these behaviors decreases, potentially decreasing exposure to toxic chemicals such as lead via this route. Other sources of lead exposure via these behaviors include ingesting chipped or flaked lead-based paint, contaminated goods like jewelry, imported toys, utensils, traditional cosmetics, and cookware [34, 35].The second factor might be gastrointestinal (GI) lead absorption in children. While children's GI system is maturing, the absorption kinetics is markedly different from the adults. On average, children absorb about 40 − 50% of lead compared to 10 − 15% of adults [36].

Reports on the association between obesity and BLLs remain inconclusive. Analysis of NHANES data between 1997 to 2000 showed a steady increase in obesity rates and a decrease in BLLs among children in the US [37]. Scinicariello et al. report an inverse association between body weight outcomes and BLLs among adolescents and children [38]. Kim et al. also reported a statistically significant correlation between BMI and lead level in teeth [39]. In contrast, we observed that children and adolescents who had central obesity had significantly lower BLLs compared to their counterparts who did not. BLLs are proportional to total body lead concentration only if there is chronic exposure to lead. While BLLs have decreased consistently over the past few decades owing to environmental reforms like the ban on leaded gasoline and lead pipes, the body burden of lead may last for decades [40]. A reverse causality association between obesity and BLLs cannot be excluded, and future research must address this issue.

The study's strength includes a large nationally representative sample and follows the study design and sampling protocols. BLLs were measured using ICP-MS, a gold standard to analyze BLLs accurately [41]. In addition, serum cotinine levels, a biological indicator, were utilized to assess SHS exposure in our study, which is more reliable compared to the self-reported number of smokers in the household relying on responders' memories [42]. However, our study has several limitations. First, there is a lack of other potential confounding variables, such as environmental conditions, such as the year their residences were built, behavioral factors, and other sources of lead exposure, such as dust inhalation and consumer products, which have not been collected in the NHANES data. Second, this study includes children aged 6 to 19 whose serum cotinine and BLLs were available in the NHANES database, which means the association could be underestimated because our analysis did not include younger children aged less than 6 years who spend more time exposed to secondhand smoke and bear a higher lead exposure burden than older children. Lastly, due to the cross-sectional study design, the temporal link between SHS exposure and BLLs could not be assured.

## Conclusion

The study provides additional evidence supporting secondhand smoke may be a source of lead exposure among children and adolescents in the US. These findings have implications for lead exposure prevention programs and policy redirection. Accelerated incorporation of smoke-free initiatives should be promoted under lead poisoning prevention programs, especially among communities dwelling in environmentally depleted and low socioeconomic neighborhoods, and the focus needs to be redirected from the primary sources of lead exposure to other often neglected sources such as secondhand smoke, especially for chronic low-level lead exposure. Although implementing a public policy on a large scale might seem far-fetched, especially in private home settings, banning smoking in environments with children will help reduce the detrimental health outcomes of chronic low-level lead exposure. An actionable change includes continued education of parents about secondhand smoke as a source of lead exposure for their children that will help prevent chronic exposure to low lead levels.

## Data Availability

The data analyzed for this study are publicly available data from the National Health and Nutrition Examination Survey available at https://www.cdc.gov/nchs/nhanes/index.htm.

## Abbreviations

ACCLPP: Advisory Committee on Childhood Lead Poisoning Prevention
BLL: Blood Lead Level
BMI: Body Mass Index
CDC: Centers for Disease Control
CI: Confidence Interval
EPA: Environmental Protection Agency
GI: Gastrointestinal
GM: Geometric Means
ICP-MS: Inductively Coupled Plasma Mass Spectrometry
IQR: Interquartile Range
LOD: Limit of Detection
NHANES: National Health and Nutritional Examination Survey
PIR: Poverty Income Ratio
SHS: Secondhand smoke
USEPA: US Environmental Protection Agency
